# Parental relationship satisfaction, reflective functioning, and toddler behavioral problems: A longitudinal study from pregnancy to 2 years postpartum

**DOI:** 10.3389/fpsyg.2022.904409

**Published:** 2022-08-12

**Authors:** Saara Johanna Salo, Jari Olavi Lipsanen, Johanna Sourander, Marjukka Pajulo, Mirjam Kalland

**Affiliations:** ^1^Department of Education, University of Helsinki, Helsinki, Finland; ^2^Department of Psychology, University of Helsinki, Helsinki, Finland; ^3^Department of Child Psychiatry, University of Turku, Turku, Finland

**Keywords:** relationship satisfaction, parental reflective functioning, behavioral problems, pregnancy, toddlerhood, longitudinal relationship satisfaction, PRF, toddler behavioral problems

## Abstract

Parent relationship satisfaction and parental reflective functioning (PRF) are significant factors in the transition to first-time parenting and are likely to affect a child’s later wellbeing. However, little is known about their joint longitudinal effects from pregnancy onward. Starting in the prenatal period, this follow-up study of 1016 Finnish first-time parents (358 fathers and 658 mothers at baseline) examined the stability and the reciprocal associations between relationship satisfaction and PRF in predicting child behavioral problems (CBCL) at age 2. First, the results of the random-intercept cross-lagged panel models showed that both relationship satisfaction and PRF were stable from pregnancy onward for both mothers and fathers, with the exception of mothers’ prenatal PRF. Second, there were significant reciprocal associations between low prenatal PRF and low relationship satisfaction at age 1, and vice versa. Third, for both mothers and fathers, a low level of relationship satisfaction, but not PRF, predicted consistently higher levels of child behavioral problems at age 2. These results suggest that parent relationship satisfaction and PRF are stable but largely independent parental factors during the transition to parenthood. In addition, our results highlight the significant role of parent relationship satisfaction in predicting toddler behavior problems, which indicates the relevance of early relationship-orientated help for first-time parents.

## Introduction

Toddlerhood is a challenging developmental period that is often characterized by an increase in externalizing and internalizing behavioral problems (Egger and Angold, 2006). These early behavioral issues often occur simultaneously and are rather stable (Basten et al., 2016); in addition, they may predict later psychiatric symptoms (Bornstein et al., 2010). From the parental perspective, the first-time transition from pregnancy to parenting is likely to negatively affect a couple’s relationship satisfaction (Don and Mickelson, 2014; Doss and Rhoades, 2017), which, in turn, may serve to predispose a toddler to behavioral problems. Furthermore, in line with the family systems theory, relationship problems may influence other parenting skills (Christopher et al., 2015), and thus further affect the child’s wellbeing (Knopp et al., 2017). Parental reflective functioning (PRF) aids a parent’s capacity to consider and understand the child’s perspective and behavior, and PRF has recently been underlined as a key skill that can help parents navigate the transition to parenthood and their new roles (Slade, 2005; Camoirano, 2017). To date, however, little is known empirically about the longitudinal stability and interrelationships between relationship satisfaction and PRF as predictors of toddler behavior problems that may follow first-time parents’ transitions from pregnancy to toddlerhood.

The transition to first-time parenthood changes a parental couple’s romantic relationship. Although research has indicated that relationship satisfaction increases during pregnancy for first-time parents, most couples report a decline in relationship satisfaction during the 1st year of their infant’s life (Doss and Rhoades, 2017). Parents of infants, especially mothers, appear to have the lowest levels of satisfaction (Twenge et al., 2003) when compared to non-parents with similar relationships lengths (Lawrence et al., 2008). Nevertheless, a meta-analysis comparing new parents to childless couples identified the same decline in satisfaction in both groups, which suggests that a negative change may reflect the passing of time and not be specifically related to childbirth (Mitnick et al., 2009). It has been suggested that negative changes that are specifically related to the transition into parenthood may reflect the necessary adaptions to an altered life situation and an internalization of the new roles within the family system (Kluwer, 2010). Although this negative change could be partially viewed as adaptive and a reflection of systemic change, most couples have described their dissatisfaction as harmful because it increases the negative aspects of a relationship and decreases the positive aspects, such as affection and intimacy (Doss et al., 2009).

Consequently, it is not surprising that relationship dissatisfaction can have direct negative consequences for the child: children may learn maladaptive behaviors to protect themselves against negative emotions (Cummings and Davies, 2010) or parental conflict may alter parenting aptitude —the so-called spillover effect (Erel and Burman, 1995; Krishnakumar and Buehler, 2000). Supporting the first hypothesis, previous research has indicated that among 3-year-old children, paternal relationship dissatisfaction is directly related to the Child Behavior Checklist Dysregulation Profile (CBCL-DP), which refers to a pattern of elevated scores on the attention problems, aggression, and anxiety/depression subscales of the CBCL (Kim et al., 2012). Low satisfaction among both mothers and fathers has also been related to higher levels of child behavior problems in developmentally delayed children (Robinson and Neece, 2015). In a large clinical sample of 4–18-year-old children, Jucksch et al. (2011) found that the CBCL-DP was associated with greater familial psychopathology and additional problems with interfamilial relationships, family communication, upbringing, and the immediate environment. Likewise, relationship dissatisfaction has been associated with parent-reported child internalizing and externalizing behavioral problems (Knopp et al., 2017). Studies of younger children have also shown associations between marital conflict and increased emotional and behavioral problems (Frankel et al., 2015). However, most studies have been conducted with children who are pre-school aged or older, and less is known about the period from pregnancy to early toddlerhood.

The significant role of reflective parenting skills, i.e., the parental capacity to show coherent and appropriate appreciation of the child’s internal states, has recently been underlined in the transition to parenthood (Slade, 2005; Camoirano, 2017). The development of PRF begins prenatally as part of the preparation for parenthood (Slade et al., 2009; Pajulo et al., 2015), and studies have shown that PRF is related to other important parenting variables, such as early emotional availability toward the baby (Salo et al., 2019). However, the stability of early PRF has been less researched. Wong (2016) reported on the stability of PRF from the prenatal period to 7 months postnatally utilizing a non-clinical sample, and a previous study by the current authors researched stability between 3 and 12 months postnatally (Salo et al., 2021); however, other studies have generally been conducted in clinical populations and have focused on studying the changes related to early interventions (e.g., Pajulo et al., 2012; Suchman et al., 2017).

Reflective parents are open, curious, and interested; rather than simply responding to observable behaviors, reflective parents make accurate interpretations about their child’s internal experiences. Parents who are characterized as having low PRF seem unaware of their own or their child’s thoughts or feelings, and they overlook the emotional experiences associated with parenting (Slade, 2005). As such, PRF may have a key role in helping first-time parents to develop and navigate appropriate emotion-regulation and child-rearing strategies. Indeed, a number of studies have already linked PRF to several significant child outcomes. Higher maternal PRF has been related to fewer externalizing and internalizing problems (Ensink et al., 2017), while poor RF in children has, in turn, been associated with a range of behavior difficulties (Benbassat and Priel, 2012; Meins et al., 2013), including attention problems, social withdrawal, anxiety (Esbjørn et al., 2013; Smaling et al., 2016), and conduct issues (Sharp et al., 2006). Furthermore, Wong et al. (2017) found that PRF moderated the relationship between difficult temperaments in infants and the prevalence of behavior symptoms in toddlers. Nijssens et al. (2020) also showed that PRF moderated the association between parental attachment and child social-emotional development. Previous research has indicated that while adequate PRF can contribute positively to a child’s quality of behavior and emotion regulation, poor parental mentalizing is likely to either directly or indirectly increase the risk of a child developing behavioral problems. However, previous studies have not followed PRF longitudinally beginning prenatally; thus, less is known about the stability of the construct and its effects on behavioral problems over the early childhood period.

Family systems theory further postulates that there is a reciprocal association between the functioning in the spousal and parental subsystems. Behaviors and emotions experienced in one subsystem (e.g., relationship satisfaction) may be transferred to another subsystem (e.g., parental RF), or vice versa (Erel and Burman, 1995; Christopher et al., 2015). According to this spillover hypothesis, negative relational feelings and intra-parental conflict would lead to deficits in parenting skills. Alternatively, from a systemic perspective, poor parenting may lead to a further increase in parental conflict and an increase in relationship dissatisfaction. However, these possible reciprocal relations between relationship satisfaction and PRF are not clear. Several studies have however produced findings that support the spillover hypothesis. Luyten et al. (2017b) found that a low quality of marital relationship among both mothers and fathers was related to poor PRF. Reversely, high levels of reflectiveness are likely to increase an adult’s general capability to understand the spousal relationship during the transition into parenthood and thus increase the potential for higher relationship satisfaction. Indeed, Jessee et al. (2018) showed that higher general RF measured prenatally was later related to more positive marital quality as well as good co-parenting among mothers, but interestingly not among fathers. However, to date, relationship satisfaction and PRF have not been longitudinally assessed simultaneously from the prenatal period, and the direction of the effects has not been examined.

Early childhood behavior problems are categorized within two broad domains: externalizing and internalizing. Externalizing problems include under-controlled, acting-out behaviors, such as conduct difficulties, hyperactivity, and impulsivity. Internalizing problems involve experiences of distress (e.g., sadness/depression, worry/anxiety) that are represented by inwardly directed reactions (Achenbach and Rescorla, 2004). Longitudinal studies among normative, population-based samples have shown that about 2% of children have clinical levels of both internalizing and externalizing symptoms (Wichstrøm et al., 2012; Basten et al., 2016); other studies have suggested even higher rates for pre-schoolers, ranging from 6.7% (Sveen et al., 2013) to 14% for anxiety and depression (Egger and Angold, 2006). Even slightly elevated behavioral symptoms in early childhood can be continuous and may be systematically related to other negative outcomes in middle childhood (de Lijster et al., 2019); therefore, further studies that focus on the predictors of behavioral problems in population-based samples are warranted.

Finally, parenting literature has frequently called for the systematic inclusion of both mothers and fathers. Indeed, the few parenting studies that have produced reports from both fathers and mothers on relationship satisfaction have used either the total or average relationship satisfaction scores of both spouses (Sturge-Apple et al., 2004) or used the summed scores as manifest indicators to create a single latent construct (Kaczynski et al., 2006). In addition, many parenting studies have assessed relationship satisfaction according to a single reporter (Davies et al., 2009), despite research indicating that there may be gender-related differences in relationship satisfaction (Lawrence et al., 2008) and reflective functioning (Jessee et al., 2018). The PRF literature has also emphasized the importance of including fathers, as there could be differences in their levels of PRF and other parenting factors related to PRF, such as demographic features, attachment, and symptomatic distress (Luyten et al., 2017b; Pazzagli et al., 2018). These potential differences highlight the need to study PRF in mothers and fathers separately.

This study aimed to add to the extant research by utilizing a longitudinal data set of first-time parents to examine the stability and interrelations between relationship satisfaction, PRF, and child behavioral problems from pregnancy to 2 years postpartum. Based on previous research and theory, we first hypothesized that both relationship satisfaction and PRF measured prenatally, and at 3, 12, and 24 months postnatally would show stability over time. Second, according to the spill-over hypothesis, we predicted that relationship satisfaction and PRF would interact together. However, few empirical studies have addressed the longitudinal interrelations between relationship satisfaction and PRF; therefore, we hypothesized that the crossover effects would be indicated at an early age by the presence or absence of indirect effects from either relationship satisfaction or PRF on a child’s behavior problems at age 2, each mediated by the other. We therefore regarded these tests as exploratory. Third, we hypothesized that both relationship satisfaction and PRF would predict child behavior symptoms at age 2. Finally, as there has been little research studying PRF and relationship satisfaction in both mothers and fathers, no hypotheses were set regarding the differences in the associations between the study variables.

## Materials and methods

### Participants

The study utilized a community-based sample of pregnant first-time parents from 80 communities across Finland; the participants were invited to take part in the research *via* the system of maternity and well-baby clinics between January 2011 and October 2015 (Kalland et al., 2016). The current research was part of the Families First study, a large study on first-time parents in Finland, which followed a total of 1016 first-time parents-to-be from pregnancy until the child was approximately 2 years old; the data gathering phase included five different study points and the option of later follow-ups. The scientific goal of the Families First study was to research the wellbeing of children and their parents and investigate how the current transition to becoming a parent (mother or father) in Finland is affected by previous childhood experiences, by relationship status and quality, and by informal and formal social support. In the current study, we investigated the first (prenatal), third (3 months), fourth (12 months), and fifth (24 months) study points. The second study point (done at 1 months) involved asking missing sociodemographic data, and did not include any measures of the present study and was therefore not used in the present study. The ethical committee of the Finnish National Institute approved the study plan for health and wellbeing.

In Finland, all mothers during pregnancy and their partners are provided cost-free services in maternity clinics within primary health care. Similarly, when the baby is born, the family can access cost-free primary health care services and health check-ups for the infant in the well-baby clinics until the child is 7 years old. The service also includes two home-visits, one at week 30–32 of pregnancy and the other 1–7 days after returning home following the delivery. According to Finnish national statistics, 99.7% of all mothers and up to 97.8% of high-risk mothers use the service during pregnancy (Kalland et al., 2006). Almost all first-time mothers and fathers are also offered cost-free antenatal classes, and most parents take part in these courses. The services at the well-baby clinics are free of charge for families, and most Finnish families go to well-baby clinics.

### Procedure

The data collection was carried out using online questionnaires, which participants accessed with a personal code. Mothers and fathers were invited to the study independently of each other and not as gamily units. Paper versions of the questionnaires were also available upon request. The public health nurses in the maternity clinics informed potential participants (i.e., parents expecting their first child) about the study at a regular visit during the third trimester of pregnancy. The average gestational week the mothers answered the first survey was 34.4 (SD 3.2). The nurses were specifically instructed not to select families for the study, but to offer the opportunity to all first-time parents during a regular check-up. Either the mother or the father, or both, received written information about the study as well as informed consent forms with a prepaid envelope. The mothers and fathers who took part in the study separately filled in the same questionnaires. All parents gave their voluntary, informed consent for treatment and were instructed that they could leave the study or treatment at any time.

### Measures

#### Sociodemographic characteristics of the sample

The background information used in the present study included parental age, marital status (single/divorced, cohabiting, married), and parental level of education. The questionnaire included a nine-point list of alternatives to assess the level of education, with a higher number indicating a higher education level. The education levels ranged from the lowest category of less than the compulsory 9 years of school to the highest category of university doctorate degree. For descriptive purposes, Table 1 includes the following four categories of education: (1) basic level = compulsory school of 9 years; (2) vocational 2nd degree = professional training, high school; (3) vocational 3rd degree = polytechnic degree; and (4) high = academic (basic or research degree).

**TABLE 1:** Participants’ socio-demographic characteristics at baseline.

	Mothers		Fathers	
		
	*M*	*SD*	*M*	*SD*
Age	29.17	4.62	30.94	4.91
	*n*	%	*n*	%
Level of education				
Only basic education	22	3.7	24	8.7
Vocational 2nd degree	155	25.8	100	36.2
Vocational 3rd degree	215	35.8	73	26.4
Academic	208	34.7	79	28.6
Matrimonial status				
Married	303	50.5	161	58.3
Cohabiting	278	46.3	112	40.6
Single	19	3.2	3	1.1

#### Index of marital satisfaction

To assess relationship satisfaction, we used the Index of Marital Satisfaction (IMS) (Hudson, 1997). The IMS is a 25-item scale designed to measure a person’s degree of dissatisfaction with their relationship. The item scores range from 1–7; thus, a theoretical total score is between 25 and 175. A higher score indicates more dissatisfaction in the relationship. The IMS has been found to have good internal consistency (alpha.96) and concurrent, construct, and discriminant validity (Hudson, 1997; Torkan and Moulavi, 2009). A score of 70 or over indicates serious problems in the relationship. In this study, estimates of internal consistencies ranged between α = 0.95−0.97 for mothers and between α = 0.96−0.97 for fathers at the different study points.

#### Prenatal parental reflective functioning questionnaire

The P-PRFQ (Pajulo et al., 2015) consists of 14 statements that require the participant to rate concepts relating to their child that reflect the three P-PRFQ dimensions: (1) opacity of mental states (e.g., “I think I will always know what my child wants”); (2) reflecting on the fetus/child (e.g., “I often wonder what the baby expects and needs from me now while I am pregnant”); and (3) the dynamic nature of the mental states (e.g., “Nowadays I often try to imagine which moments with the baby will be most difficult for me after he/she is born”). Each statement is rated using a 7-point Likert-type scale from “1 - strongly disagree” to “7 - strongly agree.” Optimal reflective functioning is indicated by higher scores for the nine items for reflecting on the fetus/child and the dynamic nature of the mental states as well as the one item scored in reverse. For the four items in the opacity dimensions, the middle point is considered the optimal point (4 = *optimal PRF*, 1 and 7 = *low PRF*; i.e., scoring is 1, 3, 5, 7, 5, 3, 1). In this study, we used the total sum score comprising all the subscales. Internal consistency for the total PRFQ (Cronbach’s alpha) was α = 0.90 for mothers and α = 0.88 for fathers.

#### Parental reflective functioning questionnaire-fi

To assess PRF, we used the Finnish shortened version of the original Parental Reflective Functioning Questionnaire (PRFQ; Luyten et al., 2017b) called the PRFQ-Fi (Pajulo et al., 2018). In a large population-based study of Finnish infants by Pajulo et al. (2018), exploratory and confirmatory factor analysis resulted in the 14-item PRFQ-Fi in which four factors capture the relevant aspects of early PRF: (1) interest and curiosity in the child’s individual mental states (5 items; e.g., “I am often curious to find out how my baby feels”); (2) understanding the opaque nature of mental states (3 items; e.g., “My child can react to a situation very differently than I think he/she will”); (3) appropriateness of reasoning about the mental states underlying the child’s reactions (3 items; e.g., “I hate it when my baby cries and/or seeks my attention just when I am on the phone with someone”); and (4) acknowledging the uncertainty in interpreting the child’s mental states (3 items; e.g., “I always know why my baby reacts the way he/she does”). Each scale is rated on a 7-point Likert-scale ranging from “1 - strongly disagree” to “7 - strongly agree.” Before any statistical analysis, the scores of the answers in the current study were converted according to the scoring key so that the responses were all in the same direction (higher score indicating higher mentalization). We also used the total sum score comprising all the subscales. Internal consistencies for the total PRFQ (Cronbach’s alpha) ranged between α = 0.95−0.92 for mothers and between α = 0.88−0.92 for fathers at the different study points.

#### The child behavior checklist

The CBCL (Achenbach and Edelbrock, 1991) was used to assess internalizing and externalizing behavioral symptoms. The 113-item checklist is designed to quantify a broad range of clinically relevant behavioral and emotional problems. Internalizing symptoms comprise issues such as withdrawal, somatic complaints, anxiety, and depression, while externalizing symptoms comprise delinquent, restless, aggressive, and oppositional behaviors. Parents estimate the degree or frequency of each behavior in their child on a 3-point scale: 0 (not true), 1 (somewhat or sometimes true), or 2 (very true or often true). Scores are then summed and converted to T-scores (*M* = 50, *SD* = 10) on seven different syndrome scales (emotionally reactive, anxious/depressed, somatic complaints, withdrawn, sleep problems, attention problems, and aggressive Behavior), as well as five different DSM-oriented scales (affective problems, anxiety problems, pervasive developmental problems, attention deficit/hyperactivity problems, and oppositional defiant problems). These scores combine to yield an internalizing problems, externalizing problems, and total problems composite score, and this method was used in the present study. The manual for the CBCL reports adequate reliability and validity for the scale scores (Achenbach and Edelbrock, 1991); the CBCL has also been validated and tested with Finnish child psychiatric samples (Sourander and Piha, 1997). Internal consistency for the total CBCL score (Cronbach’s alpha) was b α = 0.95 for mothers and α = 0.92 for fathers.

### Data analysis strategy

The preliminary analyses for the means, standard deviations, and bivariate correlations of the study variables were performed using the SPSS version 25. We determined whether there were differences between mothers and fathers in the background variables using Student’s *t*-tests or chi-square tests (depending on whether the variable was continuous or categorical). Descriptive statistics were then conducted to evaluate the distribution, mean, and standard deviation (SD) of each study variable, and comparisons were made to assess whether there were differences between mothers and fathers (Student’s *t*-tests). We also conducted bivariate correlations (Pearson’s r) between the study variables. We applied structural equation modeling to the analyses of the temporal associations between PRF, relationship satisfaction, and CBCL. We used random intercept cross-lagged panel data modeling (RI-CLPM) with the lavaan R-package (Rosseel, 2012), as this method allows for the inclusion of the within-subject level in the model, unlike the traditional cross-lagged modeling (Hamaker et al., 2015). To increase the stability of the models, cross-lagged paths were constrained to be equal in all models. Model fit was evaluated with the chi-square measure of exact fit, the Root Mean Square Error of Approximation (RMSEA) and its 95% confidence interval, the Comparative Fit Index (CFI), and the Tucker-Lewis Index (TLI). We tested three models: (1) an unconstrained model in which the associations of the parameters were allowed to vary; (2) a constrained model in which all the associations of the parameters were allowed to vary between mothers and fathers and on the basis of the results; and (3) a model in which one association (that became statistically significant between mothers and fathers in model 1) was allowed to vary between mothers and fathers. We then tested which of the models was the best fit in order to select the final model. Finally, we also tested the associations separately with using only those parents who entered the study together (*n* = 106).

## Results

### Participant characteristics

The vast majority of the participants were married or cohabiting (96.8% of mothers and 98.9% of fathers), were between the ages of 17 and 49 (for mothers *M* = 29.17, *SD* = 4.62 and for fathers *M* = 30.94, S*D* = 4.91), and had received an education beyond the 9 years of compulsory school (96.3% of mothers and 91.2% of fathers). Mothers and fathers differed in terms of age, *t*(844) = −5.09, *p* < 0.01, and educational level, χ^2^(3) = 23.29, *p* < 0.001, with mothers being younger and more educated than fathers. There was no difference in the matrimonial status, χ^2^(4) = 7.04, ns (Tables 1, 2). Children were born mostly full-term (gestational week *M* = 39.7 *SD* = 1.76), with birth weight *M* = 3441.5 (*SD* = 495), and Apgar *M* = 8.54 (*SD* = 1.47).

**TABLE 2:** Means and standard deviations of the study variables for mothers and fathers.

Variables relationship satisfaction prenatal	Mothers		Fathers					
		
	*M*	*SD*	*M*	*SD*	*t*-test (*df*)	*p*	*D*	*Range*
	11.67	10.57	10.85	9.50	1.07 (799)	ns	0.08	0−76
Relationship Satisfaction 3 months	13.10	11.69	12.79	1.17	0.33 (780)	ns	0.03	0−87
Relationship Satisfaction 12 months	16.54	14.52	16.67	14.93	−0.102 (637)	ns	−0.01	0−84
Relationship Satisfaction 24 months	17.52	14.99	18.38	16.18	−0.51 (453)	ns	−0.06	0−80
PRF Prenatal	4.34	0.82	4.00	0.76	5.63 (807)	<0.001	0.42	1−6
PRF 3 months	5.07	0.37	5.00	0.36	2.51 (797)	<0.01	0.19	3−6
PRF 12 months	5.06	0.36	4.99	0.37	2.11 (653)	<0.05	0.19	4−6
PRF 24 months	5.05	0.34	4.98	0.36	1.67 (461)	ns	0.18	4−6
Child Behavioral Problems 24 months	16.31	10.06	14.85	9.99	1.32 (461)	ns	0.15	0−67

### Associations of background and study variables

We then examined the associations between the participants’ background characteristics (educational level, relationship status), and the study variables (relationship satisfaction, PRF, and CBCL respectively) using Student’s t-tests. We also examined the association between child birth characteristics and study variables using correlations. For the purposes of these analyses, categorical variables (educational level, relationship status) were dichotomized to maintain adequate cell sizes (lower/higher educational level, married/cohabiting parent). The number of single parents was very low (*N* = 22). The data was checked to ensure that there were no statistical differences between the three groups (married, cohabiting, single) in relation to the other background or study variables; the participants who were single parents were then excluded from the analyses to keep the group sizes equal. The results indicated associations with a higher educational level and PRF in all the different study phases for mothers and at 3 and 12 months for fathers (Table 3). Additionally, among mothers, a higher educational level was associated with a lower level of child behavioral problems at 24 months. Relationship satisfaction was unrelated to educational status in all the study phases for both mothers and fathers. Among mothers, being married (vs. cohabiting) was associated systematically with a higher relationship satisfaction, with the exception of age 2, and also with higher PRF beyond the first study phase; the marital status of mothers was not related to child behavioral symptoms. Among fathers, being married was related to a higher relationship satisfaction prenatally and higher PRF at 3 and 12 months; the marital status of fathers was not related to child behavioral symptoms. Child birth characteristics were unrelated to the study variables (Table 4).

**TABLE 3:** Associations between the background and study variables for mothers and fathers.

Background and study variables	Educational level (high/low)^a^						Marital status (married/cohabiting)					
			
	Mothers			Fathers		Mothers			Fathers			
				
	*t* (*df*)	*P*	*D*	*t* (*df*)	*p*	*d*	*t* (*df*)	*p*	*d*	*t* (*df*)	*p*	*d*
Relationship Satisfaction Prenatal	−1.50 (524)	ns	0.14	−1.35 (273)	ns	−0.16	3.98 (515)	<0.001	0.35	2.16 (270)	<0.05	0.27
Relationship Satisfaction 3 months	−1.71 (506)	ns	0.18	−0.34 (183)	ns	−0.51	3.84 (499)	<0.001	0.34	0.78 (181)	ns	0.13
Relationship Satisfaction 12 months	−1.48 (436)	ns	0.27	−1.18 (138)	ns	−0.22	2.72 (449)	<0.01	0.261	1.96 (136)	<0.05	0.36
Relationship Satisfaction 24 months	−1.37 (323)	ns	0.18	−0.36 (88)	ns	0.08	1.70 (318)	ns	0.19	0.39 (86)	ns	0.09
PRF Prenatal	2.15 (531)	<0.05	−0.24	1.09 (274)	ns	−0.13	0.74 (515)	ns	−0.07	0.89 (271)	ns	0.11
PRF 3 months	3.62 (519)	<0.001	−0.35	4.52 (184)	<0.001	−0.68	−3.06 (504)	<0.01	−0.27	−3.59 (182)	<0.001	−0.56
PRF 12 months	2.31 (448)	<0.01	−0.25	2.91 (139)	<0.01	−0.52	−2.53 (436)	<0.05	−0.24	−0.73 (137)	ns	−0.13
PRF 24 months	2.59 (329)	<0.01	−0.33	0.95 (88)	ns	−0.21	−2.14 (322)	<0.05	−0.24	−0.29 (86)	ns	0.07
Child Behavioral Problems 24 months	2.18 (329)	<0.05	0.28	0.75 (88)	ns	0.17	−0.78 (322)		ns	0.104 (86)	ns	0.03

^a^Educational Level High, Polytechnic and academic degree; Educational Level Low, Compulsory school and professional/high school.

**TABLE 4:** Correlations between the child characteristics and the study variables for mothers and fathers.

	Gestational weeks		Birth weight		Apgar	
			
	Mothers	Fathers	Mothers	Fathers	Mothers	Fathers
(1) Relationship Satisfaction, Prenatal	−0.061	−0.032	−0.021	−0.011	−0.021	−0.001
(2) Relationship Satisfaction, 3 months	−0.076	−0.054	−0.012	0.003	−0.002	−0.034
(3) Relationship Satisfaction, 12 months	−0.045	−0.024	−0.101	−0.023	−0.001	−0.027
(4) Relationship Satisfaction, 24 months	−0.087	−0.061	−0.013	−0.001	−0.023	−0.005
(5) PRF, Prenatal	0.072	−0.057	−0.086	−0.012	0.058	0.006
(6) PRF, 3 months	0.056	0.019	0.008	−0.018	0.006	−0.001
(7) PRF, 12 months	0.055	0.062	0.055	0.021	0.091	0.114
(8) PRF, 24 months	0.117	0.008	0.006	0.059	0.054	0.082
(9) Child Behavioral Problems, 24 months	−0.101	−0.007	−0.001	−0.091	−0.092	−0.065

### Correlations between the study variables

Significant small to large size correlations (Tables 5, 6) were found between the study variables for both mothers’ and fathers’ scores, revealing that the scores between the study variables covary. Both the IMS and PRF showed consistently significant autocorrelations over time, with correlations ranging from Pearson’s *r* = 0.70−0.85 for IMS, and *r* = 0.12−0.75 for PRF across the time points. The IMS and PRF also showed significant correlations with each other over time, but only for mothers. For fathers, a high level of relationship satisfaction was correlated with lower PRF prenatally and at 3 months (*r* = −0.13−0.24). Only the IMS showed significant correlations with child behavioral symptoms, *r* = 0.14−0.28 for both mothers and fathers.

**TABLE 5:** Correlations between the study variables for mothers.

	1	2	3	4	5	6	7	8	9
(1) Relationship Satisfaction, Prenatal		0.741***	0.702***	0.631***	−0.015	−0.072	0.055	−0.045	0.139*
(2) Relationship Satisfaction, 3 months	0.741***		0.700***	0.632***	−0.021	−0.057	0.019	−0.082	0.182***
(3) Relationship Satisfaction, 12 months	0.702***	0.700***		0.780***	0.020	−0.086	0.008	−0.055	0.147**
(4) Relationship Satisfaction, 24 months	0.631***	0.632***	0.780***		0.022	−0.117*	−0.017	−0.019	0.191***
(5) PRF, Prenatal	−0.015	−0.021	0.020	0.022		0.224***	0.221***	0.180**	0.052
(6) PRF, 3 months	−0.072	−0.057	−0.086	−0.117*	0.224***		0.664***	0.639***	−0.008
(7) PRF, 12 months	0.055	0.019	0.008	−0.017	0.221***	0.664***		0.712***	−0.037
(8) PRF, 24 months	−0.045	−0.082	−0.055	−0.019	0.180**	0.639***	0.712***		0.007
(9) Child Behavioral Problems, 24 months	0.139*	0.182***	0.147**	0.191***	0.052	−0.008	−0.037	0.007	

***Correlation is significant at the level p < 0.001; **Correlation is significant at the level of p < 0.01; *Correlation is significant at the level of p < 0.05.

**TABLE 6:** Correlations between the study variables for fathers.

	1	2	3	4	5	6	7	8	9
(1) Relationship Satisfaction, Prenatal		0.851***	0.665***	0.716***	−0.131*	−0.195**	−0.103	0.006	0.315**
(2) Relationship Satisfaction, 3 months	0.851***		0.749***	0.788***	−0.138	−0.244***	−0.148	−0.050	0.279**
(3) Relationship Satisfaction, 12 months	0.665***	0.749***		0.893***	−0.047	−0.157	−0.058	0.006	0.159
(4) Relationship Satisfaction, 24 months	0.716***	0.788***	0.893***		−0.054	−0.121	−0.006	−0.001	0.196*
(5) PRF, Prenatal	−0.131*	−0.138	−0.047	−0.054		0.191**	0.122	0.140	0.090
(6) PRF, 3 months	−0.195**	−0.244***	−0.157	−0.121	0.191**		0.620***	0.579***	−0.161
(7) PRF, 12 months	−0.103	−0.148	−0.058	−0.006	0.122	0.620***		0.747***	0.108
(8) PRF, 24 months	0.006	−0.050	0.006	−0.001	0.140	0.579***	0.747***		0.096
(9) Child Behavioral Problems, 24 months	0.315**	0.279**	0.159	0.196*	0.090	−0.161	0.108	0.096	

***Correlation is significant at the level p < 0.001; **Correlation is significant at the level of p < 0.01; *Correlation is significant at the level of p < 0.05.

### Models for predicting child behavioral problems

**FIGURE 1:**
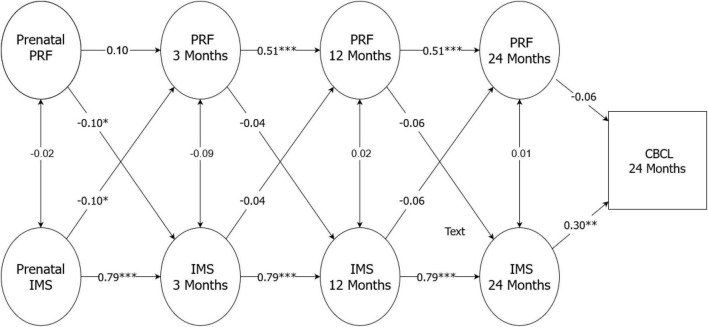
Final model for random intercept cross-lagged models for mothers.

**FIGURE 2:**
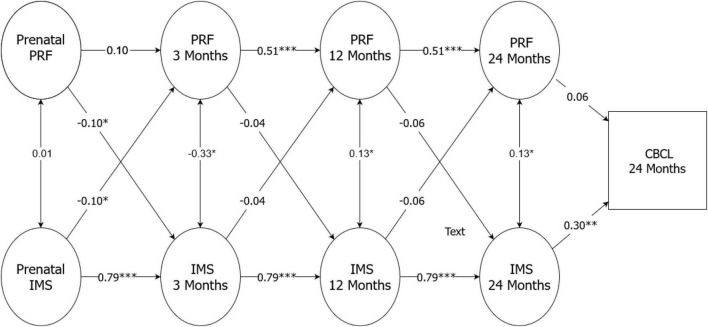
Final model for random intercept cross-lagged models for fathers.

Based on the results of the random intercept cross-lagged models, the model that showed the best fit (Table 7) was chosen as the final model. In this model, all the parameters except one (from pre- to postnatal PRF) were constrained as equal for both mothers and fathers [x^2^(55) = 66,65, RMSEA = 0.000 (0.035), CFI = 1.00 (0.983), TLI = 1.020 (0.978)]. With the exception of mothers’ pre- to postnatal PRF, both relationship satisfaction and PRF showed stability across time for both mothers and fathers. Furthermore, there were significant cross-related associations between a low level of prenatal relationship satisfaction and low PRF at 3 months, and vice versa. Finally, a low level of relationship satisfaction, but not PRF, was associated with child behavioral problems at 2 years of age. Additionally, with only using a subsample of those parents who entered the study together (*n* = 106 parents), the results showed a similar pattern of associations (Figures 1, 2).

**TABLE 7:** Comparison of the three random-intercept cross-lagged panel models.

		*df*	Chi-square	Chi-square_difference	df_difference	Pr (>Chi-square)	
Model 1 vs. Model 3	Model 1	39	14,746				
	Model 3	55	38,38	22,434	16	0,1297	
Model 1 vs. Model 2	Model 1	39	14,746				
	Model 2	56	56,696	33,27	17	0,01042	*
Model 2 vs. Model 3	Model 2	55	38,38				
	Model 3	56	56,696	20,34	1	6,48E−06	***

Model 1, All parameter associations were allowed to vary among mothers and fathers; Model 2, All parameter associations were identically fixed among mothers and fathers; Model 3, One association was freed among mothers and fathers. ***Correlation is significant at the level p < 0.001; **Correlation is significant at the level of p < 0.01; *Correlation is significant at the level of p < 0.05.

## Discussion

The primary aim of this study of first-time Finnish parents was to examine the stability and interrelationships of relationship satisfaction and PRF in predicting child behavioral problems from pregnancy until 2 years postpartum. With one exception for the mothers, the main results supported the hypothesis that both relationship satisfaction and PRF were highly stable from pregnancy onward, although they were largely independent from each other. However, a low level of relationship satisfaction in the prenatal period was found to be associated with low PRF when the child was 3 months, and vice versa. Finally, in terms of predicting child behavioral problems at 2 years of age, only parental relationship satisfaction was shown to be a significant predictor.

The main findings first showed that overall both relationship satisfaction and PRF were highly stable for both mothers and fathers across the follow-up period from pregnancy until 2 years postpartum. The result regarding the stability of relationship satisfaction is consistent with previous follow-up studies during infancy (Twenge et al., 2003), whereas the stability of PRF has not been studied to any significant extent among low-risk populations. Most couples report a decline in relationship satisfaction during the 1st year of their infant’s life (Doss and Rhoades, 2017); childless couples with similar lengths of relationship have reported less of a decline (Lawrence et al., 2008). The results of our study indicated that the level of relationship satisfaction was highly stable from pregnancy onward. However, when examining the mean levels of relationship satisfaction, our results showed that the mean levels of dissatisfaction among our sample of first-time parents was not very high in terms of the clinical cut-off used to identify a more severe level of dissatisfaction (Torkan and Moulavi, 2009). This result could be influenced by the generally high education levels of our sample and the characteristics of a non-clinical, normative population. There might also be other measures tapping more clearly aspects of relational satisfaction, especially among normative populations (Funk and Rogge, 2007). When examining the transition to parenthood in clinical populations, other studies have typically observed a much higher level of dissatisfaction (Odinka et al., 2018). It is perhaps positive to note that Finnish first-time parents appear to stay relatively satisfied in their relationships over the 1st years of their child’s life. Nevertheless, from a preventive perspective, our results also suggest that it would be important to identify first-time couples who have a high level of dissatisfaction, even prenatally, as the dissatisfaction is likely to continue throughout the early childhood period.

Second, our results add to previous studies on PRF by showing that parental reflectiveness, which begins to develop prenatally, was generally stable throughout early childhood. The one exception was that mothers’ prenatal PRF did not predict later PRF. Only a few longitudinal follow-up studies of PRF that were started prenatally in normative samples have shown consistency up to 7 months of age (Wong, 2016). However, prenatal reflectiveness is inherently different from postnatal reflectiveness because it relies more on the parent’s imaginative capabilities regarding future parenting rather than their ability to accurately relate to the infant’s behaviors and underlying mental states (see Slade et al., 2009; Pajulo et al., 2015). In comparison to the father’s experience, the mother’s formation of prenatal representations and images of future parenthood may be different to the actual experience of taking care of the baby once it is born (Pajulo et al., 2015). Mothers are most often the primary caregiver of the young infant and, as a result, they may be more likely to develop the skills required to accurately read the infant’s cues. It may be that for mothers, PRF is a relationship-specific skill that changes over time, while fathers may have a more general RF capacity that remains relatively stable. Indeed, prenatal PRF does not appear to be correlated between mothers and fathers from the same family unit (Pajulo et al., 2015). In addition, previous studies have found that fathers score lower in PRF, both pre- and postnatally, up to approximately 1 year postpartum (Pajulo et al., 2015; Salo et al., 2021); this finding was also observed in the present study. Once again, this may be related to the different roles of fathers and mothers in the early months of the infant’s life. Nevertheless, postnatally, parental reflectiveness is a skill that potentially helps parents navigate their new roles by enhancing their understanding of the infant’s behavior and their actual needs and wants (Slade, 2005). The present results extend previous findings by indicating that there is a high level of stability across a child’s first 2 years, which suggests from a clinical preventive perspective that parents with an initial low level of PRF would benefit from additional support. However, in our normative sample of first-time parents, the level of PRF identified *via* the PRF questionnaires developed for pregnancy (Pajulo et al., 2015) and the postnatal period (Luyten et al., 2017b; Pajulo et al., 2018) was generally rather high, indicating that first-time Finnish parents often have good reflective skills.

Interestingly, our results only partially supported our hypothesis regarding a family theoretical spill-over model in which parenting and parent relational systems influence one another. Our results showed significant cross-lagged interrelationships between a low level of prenatal relationship satisfaction and a low level of PRF at 3 months, and vice versa. This interrelation is in line with previous studies, indicating that the following spillover process seems to occur: higher levels of satisfaction experienced in the relationship influence better PRF skills in the long term (Luyten et al., 2017b). It is possible that the positive experiences of relationship satisfaction may act as a buffer against stress and help parents maintain an insightful and curious stance toward their infant’s mental states. However, our results indicated that this interrelationship could also work in the reverse direction. As such, our results support the findings of Jessee et al. (2018) that showed that prenatal RF measured from the AAI interview predicted more positive marital qualities later on. The ability to be reflective, open-minded, and curious about the child’s experience may demonstrate a more general reflective capability in human relationships. Thus, parents who have higher PRF prenatally may also be more capable of understanding their relationship with their partner, which could lead to a higher level of relationship satisfaction. Nevertheless, mentalizing for one’s partner is inherently different to the process of mentalizing for one’s child (Holmes and Slade, 2018). Notably, at least one study has supported this view by showing that, counterintuitively, partner RF (directly measuring the capacity to reflect on the thoughts and feelings of one’s spouse) has been related to a greater decrease in couple satisfaction in mothers during the transition into parenting (Borelli et al., 2020); thus, further studies that focus on different forms of RF and relationship satisfaction in the early years of parenting are warranted. The present study identified significant interrelationships in the critical transition from the prenatal to the postnatal (3 months) phase; however, relationship satisfaction and PRF in later phases appear to operate as largely independent factors in the early parental system and provide their own unique contribution to child development.

Indeed, our results showed that in our sample of first-time parents, only parent relationship satisfaction, and not PRF, predicted significant child behavioral problems at 2 years of age. We used random intercept cross-lagged panel model (RI-CLPM) for the statistical analyses which is useful as it explicitly take into account stable between-person differences so that their autoregressive and cross-lagged paths exclusively pertain to within-person associations. This result is expected, as a large number of studies, conducted particularly among older children, have shown that relationship dissatisfaction is linked to the CBCL-DP (Jucksch et al., 2011; Kim et al., 2012; Frankel et al., 2015; Knopp et al., 2017). The negative influence of parental relationship dissatisfaction potentially affects children in several ways: a child may develop maladaptive behaviors as a form of protection against the negative emotions elicited by the parental conflict (Cummings and Davies, 2010) or parents may lack skills other than PRF in their child-rearing practices (Vafaeenejad et al., 2019). Nevertheless, our results contrast with many current findings that have indicated that PRF is significantly related to various child social-emotional skills or behavioral problems (Camoirano, 2017). When considering this discrepancy, it is important to note that our sample comprised generally well-educated first-time parents who self-reported high levels of PRF and low levels of child behavioral problems. It is possible that the association between PRF and child outcomes becomes more apparent when the deficits in PRF are more severe and/or there are higher levels of child behavior problems and the parents require more reflective skills. Thus, the association between PRF and child outcomes may be more complex and non-linear, a concept that has previously been outlined by Luyten et al. (2017a). Additionally, only certain components of PRF, such as the pre-mentalizing mode, may be significant in predicting child outcomes (e.g., Nijssens et al., 2020). Therefore, to fully understand the significance of PRF and child behavioral problems, future studies should utilize more heterogenic samples and focus separately on the different dimensions of PRF.

In the study of relationship satisfaction and PRF, it is important that researchers consider the various contextual factors, such as the SES and marital status (Rosenblum et al., 2008; Mortensen et al., 2012). Our results showed that PRF was systematically related to a higher education level, while relationship satisfaction was not. In line with previous studies, the present associations indicate that relationship satisfaction may stabilize during the transition into parenthood regardless of years of education (Mitnick et al., 2009); in contrast, PRF may be more dependent on higher education (Luyten et al., 2017a; Pajulo et al., 2018). Our study also found that being married (as opposed to cohabiting) was related to both higher relationship satisfaction and PRF; this supports previous research that observed a negative cohabitation effect on relationship satisfaction, which prevailed after controlling for various covariates (Mortensen et al., 2012). Marriage potentially provides a greater sense of security and commitment during the emotionally vulnerable period of establishing a new relationship with the first-born child, and this effect has been previously observed, particularly among fathers (Dush et al., 2014).

The first limitation of the present study relates to the use of self-reports for the assessment of all the study variables; thus, the findings may in part reflect shared method variance. The high correlations between the independent variables also make estimation vulnerable to multicollinearity, leading to an increase in the possibility of Type II error, which means that the data does not reveal significant relationships that should be present. Furthermore, questionnaires cannot provide information that is as detailed and idiosyncratic as the data gathered *via* interview-based measures, such as the PDI-RF (Slade et al., 2005). Second, overall, our sample represented parents who were socioeconomically advantaged in terms of education and relationship status. We recommend that this study is replicated with more representative cohort samples as well as participants who are clinically more vulnerable. This study utilized well-baby clinics to recruit participants; this method resulted in a sample that included more mothers than fathers, as it is generally still mothers who take their children to these clinics. Future studies should try to avoid this sampling bias and focus particular attention on recruiting fathers. Finally, further research is required to examine the interrelations of the study variables among family units using questionnaires that have been answered by both parents. It is likely that both relationship satisfaction and PRF have an impact on the spouse, causing potential positive (or negative) cycles of interaction.

Despite the limitations, the longitudinal results of the present study both confirm and extend previous findings on relationship satisfaction and PRF and their relevance for child behavioral problems across the transition into parenthood. Our study highlighted the significance of relationship satisfaction, rather than PRF, as a predictor of toddler behavioral problems; this finding suggests that supporting first-time parents’ relationships with their partners may have preventive clinical value. Furthermore, the results indicated a significant interrelationship, over a period of time, between high prenatal PRF and high relationship satisfaction at 3 months, and vice versa, which underlines the importance of focusing on both PRF and relationship satisfaction during the significant transition from pregnancy to life with a young baby. In addition, our study builds on previous research by showing that parents’ PRF and relationship satisfaction are highly stable from pregnancy to 2 years postpartum; thus, our research emphasizes the importance of extending the understanding of their role in early parenting. Further longitudinal follow-up study phases at school-age will provide additional data on the present sample; the analyses will show whether the stability of PRF and relationship satisfaction is maintained and whether they are related to a wider range of child outcomes.

## Data availability statement

The datasets presented in this article are not readily available because permission to share data was not asked from the participants. Requests to access the datasets should be directed to SS, saara.z.salo@helsinki.fi.

## Ethics statement

The studies involving human participants were reviewed and approved by the Finnish National Institute of Health and Welfare. The patients/participants provided their written informed consent to participate in this study.

## Author contributions

MK and MP contributed to the original design of the study. SS and JL analyzed the data. SS and JS wrote the manuscript. All authors contributed to the manuscript revisions and have read and approved the submitted version.
